# Prognostic Value of the Cholesterol, High-Density Lipoprotein, and Glucose Index and Remnant Cholesterol Inflammatory Index in Young Patients with Acute Ischemic Stroke

**DOI:** 10.3390/jcm15114327

**Published:** 2026-06-03

**Authors:** Jiaokun Jia, Yuliang Qin, Jiahuan Guo, Xingquan Zhao, Yanfang Liu

**Affiliations:** 1 Department of Neurology, Beijing Tiantan Hospital, Capital Medical University, Beijing 100070, China; jiajiaokun@bjtth.org (J.J.); ccmujoe@mail.ccmu.edu.cn (Y.Q.); guojiahuan@bjtth.org (J.G.); 2 China National Clinical Research Center for Neurological Diseases, Beijing Tiantan Hospital, Capital Medical University, Beijing 100070, China; 3 Research Unit of Artificial Intelligence in Cerebrovascular Disease, Chinese Academy of Medical Sciences, Beijing 100070, China; 4 Beijing Institute of Brain Disorders, Collaborative Innovation Center for Brain Disorders, Capital Medical University, Beijing 100070, China

**Keywords:** cholesterol-high-density lipoprotein–glucose index, remnant cholesterol inflammatory index, young stroke, prognosis, hemorrhagic transformation

## Abstract

**Background**: Young stroke (age ≤ 45) is a growing global health concern often driven by metabolic factors, with insulin resistance (IR) and dyslipidemia being key contributors. Novel metabolic indices, the Cholesterol–High-Density Lipoprotein–Glucose (CHG) index and the Remnant Cholesterol Inflammatory Index (RCII), have emerged as promising tools, yet their prognostic value in young stroke populations remains unexplored. **Methods**: We retrospectively analyzed 541 young stroke patients (age ≤ 45 years) between January 2019 and December 2021. The associations between CHG and RCII indices and poor functional outcomes at 90 days and discharge, infarct size, and hemorrhagic transformation were evaluated using multivariate logistic regression, restricted cubic spline (RCS) analysis, and receiver operating characteristic (ROC) curves. Subgroup, interaction, and sensitivity analyses were performed to assess the stability and robustness of the findings. **Results**: A total of 541 young stroke patients were included (median age 37 years). Patients with poor 90-day functional outcome (modified Rankin Scale ≥ 2) exhibited significantly higher CHG (5.2 vs. 5.1, *p* = 0.018) and RCII (2.8 vs. 1.7, *p* < 0.001) levels. After multivariable adjustment, both indices were independently associated with poor 90-day outcome (CHG: OR 1.31 per SD, 95% CI 1.07–1.61; RCII: OR 1.23 per SD, 95% CI 1.04–1.46) and poor discharge outcome. The highest RCII quartile exhibited more than threefold increased odds of a poor 90-day outcome (OR 3.43, 95% CI 1.90–6.20). Both indices were associated with larger lesion size, and RCII was additionally associated with hemorrhagic transformation (Q4 vs. Q1: OR 2.42, 95% CI 1.23–4.75). Restricted cubic spline analyses demonstrated no evidence of non-linearity. Adding RCII to a base clinical model significantly improved discrimination (AUC 0.645 vs. 0.564, DeLong *p* = 0.004). No significant sex interaction was observed; RCII showed significant interactions with age and TOAST subtype. **Conclusions**: Both CHG and RCII indices are independently associated with poor functional outcomes, larger infarct size, and—for RCII—hemorrhagic transformation in young patients with acute ischemic stroke. These readily calculable biomarkers may provide complementary prognostic information in this population, though their clinical utility requires further prospective validation.

## 1. Introduction

The global epidemiological landscape of cerebrovascular disease has undergone a significant shift in recent decades, marked by a paradoxical rise in stroke incidence among young adults despite improvements in overall stroke mortality [1]. Recent data from the Global Burden of Disease (GBD) 2021 study indicate that the age-standardized incidence of stroke in young adults reached approximately 757,000 cases in 2021, representing a 19.11% increase since 1990 [2]. Unlike stroke in older populations, which is often driven by degenerative vascular changes, young-onset stroke is increasingly linked to metabolic-inflammatory risk factors associated with modern lifestyles, including obesity, sedentary behavior, and early-onset metabolic syndrome [3,4].

Insulin resistance (IR) has emerged as a critical, albeit under-recognized, pathophysiological driver of ischemic stroke in young adults [5,6]. IR promotes a pro-thrombotic and pro-inflammatory environment, accelerates atherosclerosis, and impairs the neuroprotective mechanisms essential for recovery after a cerebral ischemic event [7]. The Homeostatic Model Assessment of Insulin Resistance (HOMA-IR) remains the clinical gold standard for assessing IR; however, its utility in acute care settings is severely hampered by the requirement for fasting insulin measurements, high costs, and susceptibility to acute stress-related fluctuations [8,9]. Consequently, there is an urgent need for accessible, routine laboratory-derived markers that can reflect the complex interplay between lipid metabolism, glycemic control, and systemic inflammation.

Recently, two novel composite indices have gained attention: the Cholesterol–High-Density Lipoprotein–Glucose (CHG) index and the Remnant Cholesterol Inflammatory Index (RCII). The CHG index integrates total cholesterol, fasting blood glucose, and high-density lipoprotein cholesterol to capture the synergistic effects of hyperlipidemia and hyperglycemia on vascular health [10]. Preliminary evidence from large-scale cohorts, such as the China Health and Retirement Longitudinal Study (CHARLS) database, suggests that the CHG index is a more sensitive indicator of cardiovascular and cardiometabolic disease risk [10,11]. Similarly, the RCII, which combines remnant cholesterol (RC) with C-reactive protein (CRP), has demonstrated significant prognostic value in stroke risk and stroke outcomes [12,13]. Despite these promising findings, critical knowledge gaps remain. Most existing studies have predominantly focused on older populations, leaving the applicability of these indices to young stroke patients largely uncertain. Furthermore, direct comparative analyses evaluating the relative discriminative performance of different composite indices, such as CHG and RCII, for stroke outcomes are currently lacking.

Given the rising burden of young stroke and the potential of these novel markers to reflect multifaceted metabolic dysfunction, this study aims to evaluate the association between CHG and RCII indices and multiple clinical outcomes in a specialized database of young stroke patients. We hypothesized that elevated levels of these indices would be independently associated with poor functional outcomes, larger infarct size, and increased risk of hemorrhagic transformation. Furthermore, we sought to compare the discriminative performance of these two indices and explore potential effect modifications across clinically relevant subgroups in this high-risk population.

## 2. Materials and Methods

### 2.1. Ethics and Study Population

This retrospective study was conducted at the Department of Neurology, Beijing Tiantan Hospital, Capital Medical University. The study protocol was approved by the Ethics Committee of Beijing Tiantan Hospital, and the requirement for informed consent was waived due to the retrospective nature of the analysis.

Consecutive young patients with acute ischemic stroke admitted between January 2019 and December 2021 were screened for eligibility. The inclusion criteria were (1) age 18–45 years; (2) diagnosis of ischemic stroke confirmed by neuroimaging (computed tomography or magnetic resonance imaging); and (3) admission within 3 days from symptom onset. Patients were excluded if they met any of the following criteria: (1) cerebral venous thrombosis, iatrogenic stroke, or transient ischemic attack; (2) missing laboratory test results required for index calculation; (3) pre-stroke disability (modified Rankin Scale score ≥ 2); and (4) loss to follow-up or missing 90-day mRS data.

### 2.2. Data Collection

Demographic characteristics, clinical features, and laboratory parameters were systematically extracted from the database. The baseline variables collected at admission included age, gender, body mass index (BMI), smoking status, and alcohol consumption. Medical comorbidities were documented, comprising hypertension, diabetes mellitus, dyslipidemia, coronary heart disease (CHD), atrial fibrillation, pulmonary infection, and previous ischemic stroke. The indices were subsequently calculated based on their established formulas. The National Institutes of Health Stroke Scale (NIHSS) score on admission and the Trial of Org 10,172 in Acute Stroke Treatment (TOAST) etiological classification were obtained from the electronic medical record system. Blood samples for laboratory assays were collected within one hour of emergency department arrival.

### 2.3. Exposure and Outcome

The CHG index was calculated using total cholesterol (TC), fasting blood glucose (FBG), and high-density lipoprotein cholesterol (HDL-C) as follows: CHG = ln [TC (mg/dL) × FBG (mg/dL)/(2 × HDL) (mg/dL)]. The RCII index was calculated using RC and CRP, where RC is derived by subtracting HDL-C and low-density lipoprotein cholesterol (LDL-C) from TC: RC = TC (mg/dL) − HDL-C (mg/dL) − LDL-C (mg/dL), and the index is defined as RCII = [RC (mg/dL) × CRP (mg/L)]/10.

Neuroimaging was performed within 48 h of admission, and the findings were evaluated independently by two neurologists. Neurological functional dependence was evaluated using the modified Rankin Scale (mRS). Given that young stroke patients typically present with milder neurological deficits yet face substantial socioeconomic consequences, a poor functional outcome was defined as a 90-day mRS score ≥ 2. A sensitivity analysis using an mRS threshold of ≥3 was also performed. The clinical outcomes recorded in this study included poor outcome at 90 days, poor outcome at discharge, the occurrence of hemorrhagic transformation, and lesion size. Hemorrhagic transformation was classified according to the European Cooperative Acute Stroke Study (ECASS) criteria into hemorrhagic infarction (HI1, HI2) and parenchymal hematoma (PH1, PH2) subtypes. The use of reperfusion therapy and antithrombotic medication exposure among patients with hemorrhagic transformation was recorded. Lesion size was categorized as an ordinal variable based on the maximum diameter of the infarct: small infarct (<1.5 cm), medium infarct (1.5 to 5 cm), and large infarct (>5 cm). The 1.5 cm threshold was adopted based on the established radiological criterion for distinguishing lacunar from non-lacunar infarcts as defined in the TOAST classification [14], while the 5.0 cm threshold is consistent with those used in previous studies on stroke outcomes [15].

### 2.4. Statistical Analysis

Continuous variables are presented as means with standard deviations (SDs) or medians with interquartile ranges (IQRs), depending on the normality of their distribution. The normality of the data was assessed using the Kolmogorov-Smirnov test. Categorical variables are expressed as frequencies and corresponding percentages (%). Baseline characteristics between the good outcome and poor outcome groups were compared utilizing the *t*-test or the Mann–Whitney U test for continuous data and the Chi-square test or Fisher’s exact test for categorical data, as appropriate.

To investigate the independent associations between the evaluated indices and the prespecified outcomes, univariate and multivariate regression models were constructed. Specifically, binary logistic regression was employed for the binary outcomes (poor outcome at 90 days, poor outcome at discharge, and hemorrhagic transformation), whereas ordinal logistic regression was utilized for the ordinal outcome of lesion size. The multivariate models were adjusted for predefined confounding variables, including age, gender, BMI, history of hypertension, history of dyslipidemia, history of diabetes mellitus, current smoking, current alcohol consumption, history of CHD, atrial fibrillation, pulmonary infection, intravenous thrombolysis treatment, previous ischemic stroke, admission NIHSS score, and TOAST classification. The indices were analyzed both as continuous variables and categorized into quartiles, with the lowest quartile serving as the reference group. For logistic regression analyses, both the CHG index and the RCII index were standardized to z-scores (mean = 0, SD = 1), and odds ratios are reported per 1-SD increment. The results are presented as odds ratios (ORs) with corresponding 95% confidence intervals (CIs).

Potential non-linear relationships between the continuous indices and the outcomes were further explored utilizing a restricted cubic spline (RCS) model with three knots after adjusting for the aforementioned confounding variables. The discriminative performance of the indices was assessed by calculating the area under the curve (AUC) derived from receiver operating characteristic (ROC) curve analyses. To evaluate the incremental discriminative value of each index, a base clinical model incorporating the aforementioned covariates was compared with a full model that additionally included the CHG or RCII index. Discrimination improvement was assessed using the DeLong test, net reclassification improvement (NRI), and integrated discrimination improvement (IDI). Furthermore, subgroup analyses were performed across various clinical stratifications to verify the stability of the associations, and interaction analyses were conducted to determine whether the effects of the indices on outcomes were modified by these prespecified variables. All statistical analyses were performed using R version 4.5.2 and Python version 3.13.7 software, and a two-sided *p*-value < 0.05 was considered to indicate statistical significance.

## 3. Results

### 3.1. Baseline Characteristics of the Study Population

A total of 541 young stroke patients were included in this analysis, categorized into a good outcome group (90 d mRS 0–1, *n* = 430) and a poor outcome group (90 d mRS ≥ 2, *n* = 111) based on their prognosis (Table 1). The median age of the overall cohort was 37 years. Compared to the good outcome group, patients with poor outcomes exhibited significantly higher values for both the CHG (5.2 vs. 5.1, *p* = 0.018) and RCII (2.8 vs. 1.7, *p* < 0.001) indices. Furthermore, this group presented with elevated C-reactive protein levels, higher admission NIHSS scores, and a greater prevalence of large-artery atherosclerosis (LAA) and undetermined etiology (UE) subtypes. Other baseline demographic and clinical variables, including body mass index, gender distribution, and the prevalence of diabetes, hypertension, and previous ischemic stroke, demonstrated no significant differences between the two groups. The CHG index followed a normal distribution, whereas the RCII index was non-normally distributed. The distributions of continuous variables are presented in Supplementary Figure S1.

### 3.2. Associations with Clinical and Imaging Outcomes

Multivariate logistic regression analysis was performed to evaluate the independent associations between the two evaluated indices and various prognostic outcomes. Table 2 illustrates that after adjustment and standardization, the continuous CHG index was significantly associated with an increased risk of poor outcome at 90 days (OR 1.31, 95% CI 1.07–1.61, *p* = 0.010) and poor outcome at discharge (OR 1.32, 95% CI 1.10–1.59, *p* = 0.003). When categorized into quartiles, patients in the highest quartile of the CHG index had a significantly elevated risk of poor 90-day outcome (OR 1.99, 95% CI 1.09–3.61, *p* = 0.025) and poor discharge outcome (OR 1.74, 95% CI 1.03–2.92, *p* = 0.037) relative to those in the lowest quartile. Furthermore, a higher continuous CHG index was associated with larger lesion size (OR 1.40, 95% CI 1.18–1.66, *p* < 0.001), though no significant association was observed regarding hemorrhagic transformation. Among patients with hemorrhagic transformation, the distributions of ECASS subtypes, reperfusion therapy use, and antithrombotic medication exposure are summarized in Supplementary Table S1.

Similarly, the continuous RCII index emerged as an independent prognostic factor for poor outcome at 90 days (OR 1.23, 95% CI 1.04–1.46, *p* = 0.016) and poor outcome at discharge (OR 1.22, 95% CI 1.04–1.45, *p* = 0.017) as detailed in Table 3. The highest quartile of the RCII index exhibited more than three-fold increased odds in the risk of poor outcome at 90 days (OR 3.43, 95% CI 1.90–6.20, *p* < 0.001) and poor outcome at discharge (OR 3.12, 95% CI 1.86–5.21, *p* < 0.001) compared to the lowest quartile. This index was also significantly associated with larger lesion size (continuous OR 1.23, 95% CI 1.04–1.46, *p* = 0.018) and an increased risk of hemorrhagic transformation in the highest quartile (OR 2.42, 95% CI 1.23–4.75, *p* = 0.011).

### 3.3. Dose-Response Relationship Between RCII and CHG Index and Outcomes

**Figure 1:**
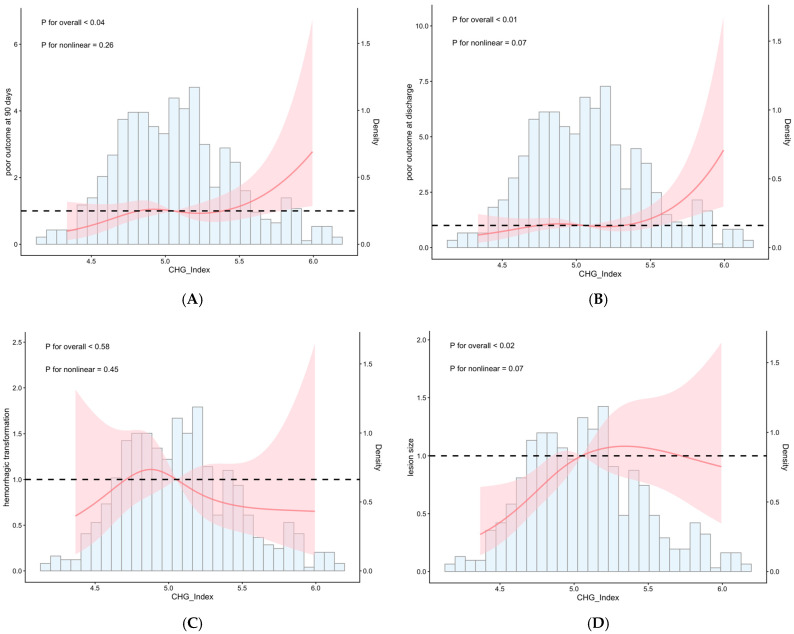
Restricted cubic spline models analyzed the relationship between the CHG index and (**A**) poor outcome at 90 days, (**B**) poor outcome at discharge, (**C**) hemorrhagic transformation, and (**D**) lesion size. CHG index, cholesterol, high-density lipoprotein, and glucose index. The underlying light blue histogram illustrates the frequency distribution of the parameter. The red solid line indicates the fitted dose-response curve, with the pink shaded area representing the 95% confidence interval. The black dashed line denotes the reference level of odds ratio equal to 1, which is anchored at the median value of the parameter.

**Figure 2:**
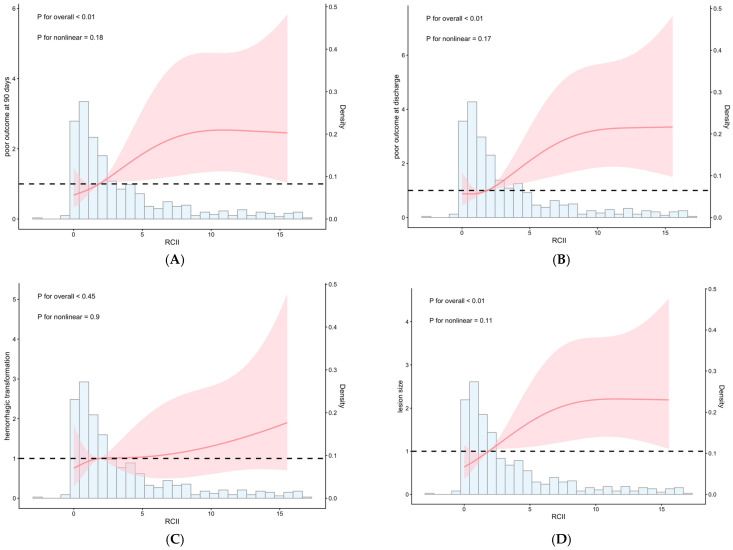
Restricted cubic spline models analyzed the relationship between the RCII index and (**A**) poor outcome at 90 days, (**B**) poor outcome at discharge, (**C**) hemorrhagic transformation, and (**D**) lesion size. RCII index, remnant cholesterol inflammatory index. The underlying light blue histogram illustrates the frequency distribution of the parameter. The red solid line indicates the fitted dose-response curve, with the pink shaded area representing the 95% confidence interval. The black dashed line denotes the reference level of odds ratio equal to 1, which is anchored at the median value of the parameter.

Figure 1 and Figure 2 illustrate the RCS curves between the CHG index, the RCII index, and the prespecified outcomes. The results revealed no evidence of non-linearity for either index across all outcomes, as all tests for non-linearity yielded *p*-values greater than 0.05, after adjusting the full model. Both indices showed significant associations with poor outcome at 90 days, poor outcome at discharge, and lesion size, with overall *p*-values < 0.05 in RCS analysis.

### 3.4. Discriminative Performance of CHG and RCII Indices

**Figure 3:**
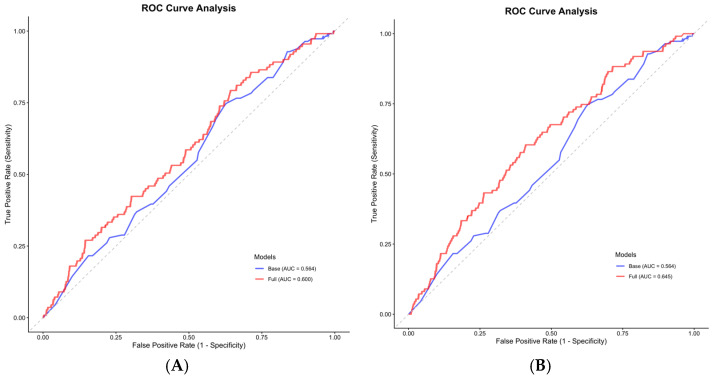
ROC curves for the base clinical model and the base model plus (**A**) CHG index and (**B**) RCII index in discriminating between patients with good and poor 90-day functional outcomes. ROC, receiver operating characteristic; CHG index, cholesterol, high-density lipoprotein, and glucose index; RCII index, remnant cholesterol inflammatory index; AUC, area under the curve. The diagonal dashed line serves as the random reference baseline (AUC 0.50).

To further explore the incremental discriminative value of the CHG and RCII indices, receiver operating characteristic curves were constructed comparing a base clinical model with a full model that additionally incorporated each index (Figure 3 and Table 4). For discriminating poor 90-day outcomes, the base clinical model yielded an AUC of 0.564 (95% CI 0.515–0.619). The addition of the CHG index modestly improved discrimination (AUC 0.600, 95% CI 0.551–0.648; NRI 0.148, 95% CI 0.065–0.234; IDI 0.008, 95% CI 0.001–0.017; DeLong *p* = 0.050). A more pronounced improvement was observed with the addition of the RCII index (AUC 0.645, 95% CI 0.595–0.695; NRI 0.286, 95% CI 0.138–0.428; IDI 0.035, 95% CI 0.020–0.049; DeLong *p* = 0.004).

### 3.5. Subgroup and Interaction Analyses

**Figure 4:**
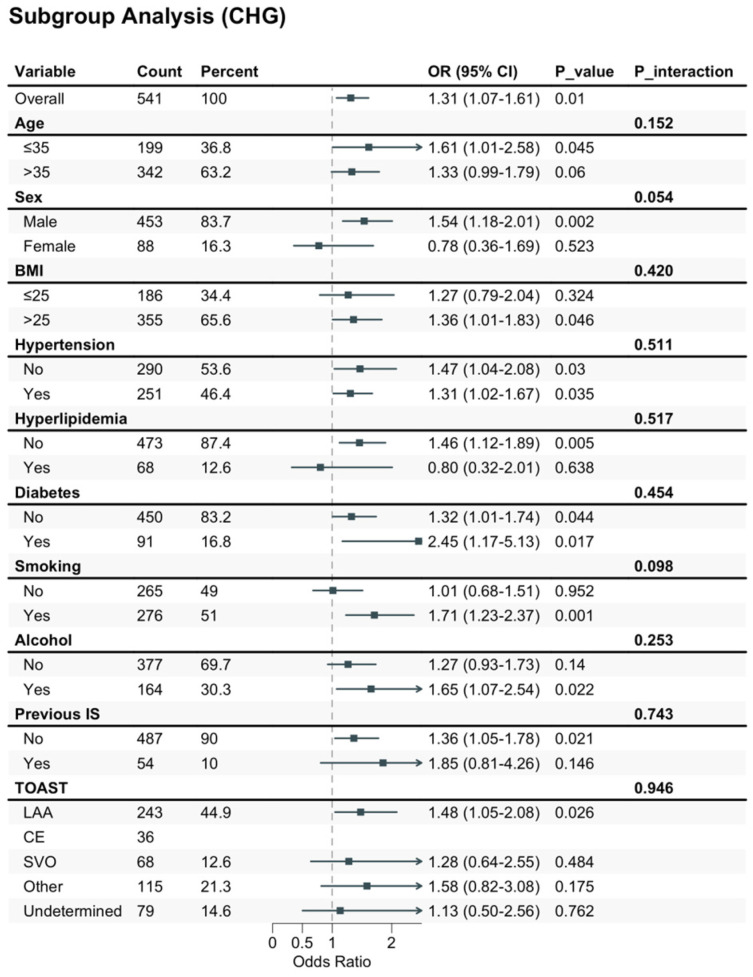
Forest plot of subgroup analysis regarding the risk associated with the CHG index and poor outcome at 90 days. The vertical dashed line represents the reference line of OR = 1; Squares represent the odds ratios for each subgroup, with horizontal lines indicating the 95% confidence intervals.

**Figure 5:**
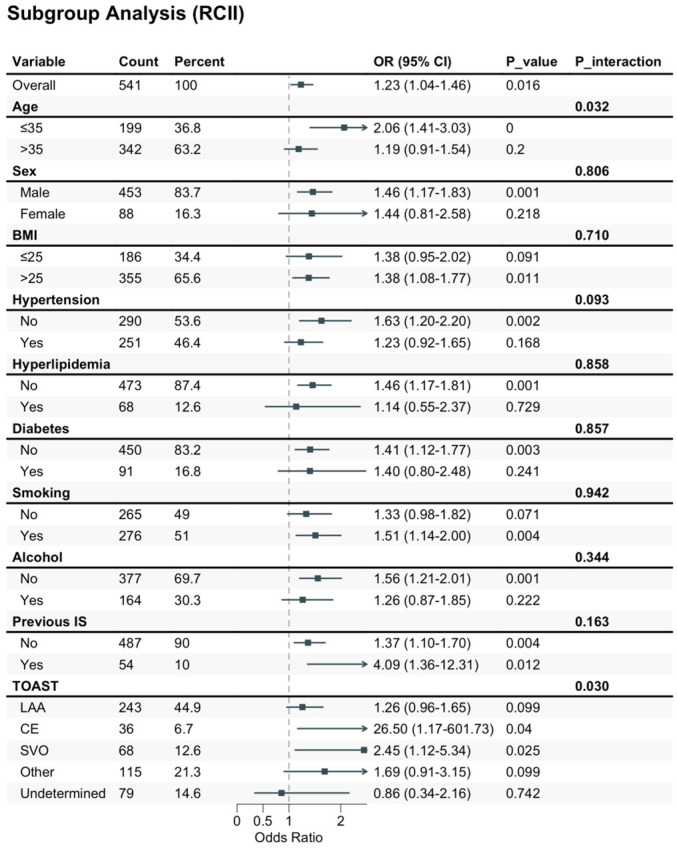
Forest plot of subgroup analysis regarding the risk associated with the RCII index and poor outcome at 90 days. The vertical dashed line represents the reference line of OR = 1; Squares represent the odds ratios for each subgroup, with horizontal lines indicating the 95% confidence intervals.

Figure 4 and Figure 5 illustrate the subgroup analyses evaluating the consistency of the associations between the indices and poor 90-day outcomes across various clinical stratifications. The association of the CHG index remained robust across all evaluated subgroups, with no significant interactions observed (all *p* for interaction > 0.05). Conversely, the RCII index exhibited a significant interaction with age (*p* for interaction = 0.032), demonstrating a stronger association with poor 90-day outcomes in patients aged ≤ 35 years (OR 2.06, 95% CI 1.41–3.03) compared to older cohorts. Furthermore, the relationship between the RCII index and poor 90-day outcomes varied significantly across TOAST etiological subtypes (*p* for interaction = 0.030).

### 3.6. Sensitivity Analysis

To test the robustness of our primary findings, we performed two sensitivity analyses: first, using a different threshold for poor outcome (mRS ≥ 3, Table 5), and second, excluding patients with pulmonary infection (Table 6).

When a poor outcome was defined as mRS 3–6 (Table 5), both CHG and RCII showed significant associations. For CHG, compared with Q1, Q2 (OR 2.50, 95% CI: 1.07–5.82, *p* = 0.034) and Q4 (OR 2.57, 95% CI: 1.02–6.45, *p* = 0.044) were independently associated with poor outcomes, although the continuous per SD and trend analyses were not significant (*p* = 0.231 and *p* = 0.143, respectively). For RCII, the association was stronger and more consistent: per SD increase (OR 1.23, 95% CI: 1.00–1.51, *p* = 0.046) and Q4 (OR 5.20, 95% CI: 2.24–12.08, *p* < 0.001) both showed significant independent associations, with a significant trend across quartiles (*p* < 0.001).

After excluding patients with pulmonary infection (Table 6), the results for RCII were consistent, with Q4 remaining significantly associated with poor outcome (OR 3.88, 95% CI: 1.56–9.66, *p* = 0.004) and a significant trend across quartiles (*p* = 0.002). For CHG, no significant associations were observed in the multivariate analysis.

## 4. Discussion

This study investigated the prognostic value of two novel metabolic–inflammatory indices—the CHG index and the RCII index—in young patients with acute ischemic stroke. Our findings demonstrate that both indices are independently associated with poor functional outcomes at 90 days and at discharge, as well as with larger lesion sizes. Additionally, the RCII index showed a significant association with hemorrhagic transformation. These results support the utility of these composite biomarkers in risk stratification following acute ischemic stroke in young adults [16,17]. Furthermore, sensitivity analyses yielded consistent findings.

Our results align with and extend previous investigations into the prognostic significance of metabolic indices in cerebrovascular disease. The CHG index integrates information about cholesterol metabolism and glucose homeostasis, two fundamental processes that are frequently disturbed in stroke patients [18,19]. Dyslipidemia and hyperglycemia promote endothelial dysfunction, oxidative stress, and prothrombotic states, thereby exacerbating ischemic injury and impeding recovery [20,21]. Previous studies have demonstrated associations between individual components of the CHG index and stroke outcomes [22,23]; however, our study is among the first to evaluate the composite index specifically in a young stroke population. The stronger associations observed with the RCII index may reflect the additional contribution of inflammation to stroke pathophysiology, as CRP captures the systemic inflammatory response that accompanies acute ischemic injury [24,25].

The observed associations between these indices and infarct size provide insights into potential mechanistic pathways. Both the CHG index (OR 1.40) and the RCII index (OR 1.23) were significantly associated with larger lesion sizes, suggesting that metabolic-inflammatory dysregulation may influence the extent of ischemic damage. Larger infarcts typically result from more severe or prolonged ischemia, greater collateral circulation failure, or heightened susceptibility to ischemia–reperfusion injury [26]. The pro-inflammatory and pro-oxidant effects of remnant cholesterol, combined with the metabolic disturbances reflected in the CHG index, may exacerbate these processes, leading to more extensive tissue damage [27,28]. The restricted cubic spline analyses revealed no evidence of non-linearity between both indices and outcomes (all *p* for non-linearity > 0.05), suggesting monotonic associations without apparent thresholds [29,30].

The pathophysiological mechanisms underlying these associations are multifaceted. Insulin resistance and dyslipidemia in young stroke patients promote a state of chronic oxidative stress and endothelial dysfunction [30]. Elevated remnant cholesterol particles can penetrate the arterial wall more easily than LDL, where they are taken up by macrophages to form foam cells, leading to unstable plaque formation and impaired collateral circulation [31]. Simultaneously, hyperglycemia promotes the formation of advanced glycation end-products (AGEs), which further aggravate vascular stiffening and neuroinflammation [32]. The synergy between these factors likely exacerbates the primary ischemic injury, resulting in the larger lesion sizes and worse functional outcomes observed in our high-CHG and high-RCII groups [33].

The association between the RCII index and hemorrhagic transformation represents a novel finding with important clinical implications. Hemorrhagic transformation occurs in approximately 10–40% of ischemic stroke patients and is associated with worse functional outcomes [34]. The pathophysiology involves disruption of the blood-brain barrier, reperfusion injury, and inflammation-mediated vascular damage [35]. The RCII index, by capturing both lipid-mediated and inflammatory pathways, may identify patients at heightened risk for this complication. Patients in the highest RCII quartile demonstrated significantly increased odds of hemorrhagic transformation, suggesting that this index may identify a subgroup of patients warranting closer observation in future prospective studies. Given the observational nature of this study, the direct implications of the index for reperfusion or antithrombotic decision-making require further investigation.

The ROC curve analyses revealed moderate discriminative performance for both indices, with AUC values ranging from 0.600 to 0.645 across different outcomes. These modest AUC values indicate limited discriminative performance, and the clinical applicability of these indices as standalone prognostic tools remains uncertain. Stroke prognosis is influenced by numerous factors, including stroke severity, time to treatment, and individual comorbidities, which collectively limit the discriminative capacity of any single biomarker [36]. The incremental value demonstrated in the present analyses should be interpreted as exploratory, and the results do not yet support the use of these indices for individual-level clinical decision-making.

Our subgroup analyses yielded important insights into the differential prognostic behavior of the two indices across clinically relevant strata. The CHG index demonstrated consistent prognostic associations across all examined subgroups without significant effect modification, suggesting that the lipid–glycemic coupling it captures reflects a broadly generalizable metabolic risk pathway that operates independently of age, sex, stroke severity, and etiological subtype.

The RCII index exhibited significant interactions with both TOAST etiological subtype and age. For TOAST subtypes, nominally significant associations were present in the cardioembolic and small artery occlusion groups, though these comprised only 36 and 68 patients, respectively. For age, a stronger association was observed in patients aged 35 years or younger compared to those aged 36 to 45 years. This suggests systemic inflammation and remnant cholesterol-mediated endothelial damage may act as primary prognostic drivers in younger populations where conventional atherosclerotic risk factors are less prevalent. The relatively homogeneous etiology in younger patients might also reduce competing risks, thereby highlighting the prognostic value of the RCII. However, given the restricted sample sizes across these specific strata, both the etiology and age interactions must be interpreted cautiously. Confirming whether etiology and age genuinely modify the prognostic role of the RCII requires validation in larger cohorts.

To assess the robustness of our primary findings, we conducted several prespecified sensitivity analyses. First, redefining poor functional outcome using a more conservative threshold (mRS ≥ 3) yielded generally consistent associations for both the CHG and RCII indices, indicating that the choice of mRS cutoff did not materially influence our conclusions. Second, excluding patients with documented pulmonary infection—whose CRP elevation might partially reflect infection rather than stroke-related inflammation—did not substantively alter the association between the RCII index and clinical outcomes, suggesting that the observed prognostic signal is not driven primarily by acute infectious processes. Collectively, these sensitivity analyses support the consistency of the associations reported in our primary models, although we acknowledge that subclinical infection and other non-infectious sources of systemic inflammation cannot be entirely excluded.

From a clinical perspective, our findings suggest that routine assessment of these indices could provide complementary prognostic information in young stroke patients. Both indices can be calculated from standard laboratory parameters obtained at admission, requiring no additional testing or specialized equipment. Early identification of high-risk patients may facilitate more intensive monitoring, prompt initiation of secondary prevention measures, and inform rehabilitation planning. The RCII index, in particular, may help identify patients at increased risk for hemorrhagic transformation who warrant closer monitoring during the acute phase.

Several limitations of this study should be acknowledged. First, the retrospective single-center design limits generalizability and may introduce selection bias. Second, the relatively modest sample size, while adequate for detecting moderate effect sizes, may have limited power for subgroup analyses and rare outcome events. Third, we did not have data on dynamic changes in these indices during the acute phase or their trajectories during recovery, which might provide additional prognostic information. Fourth, residual confounding from unmeasured variables cannot be excluded, despite adjustment for multiple established risk factors. Fifth, several clinically relevant variables were incompletely captured in the present database, including medication use (antiplatelet agents, antihypertensive therapy, and lipid-lowering drugs), baseline level of consciousness, infarct location, and detailed etiological workup data (e.g., patent foramen ovale, arterial dissection, and thrombophilia screening). The absence of these variables may have introduced residual confounding and limited our ability to fully account for etiological heterogeneity.

Future research should address these limitations through prospective multicenter studies with larger sample sizes and longer follow-up periods. Additionally, studies examining the effects of interventions targeting IR and remnant cholesterol can directly improve functional recovery in young stroke survivors. Furthermore, given the recently reported association between remnant cholesterol and renal dysfunction [37], future studies should explore whether the RCII index is associated with renal parameters such as estimated glomerular filtration rate and chronic kidney disease status, as renal dysfunction may represent a clinically relevant link between remnant cholesterol, systemic inflammation, and adverse outcomes after young stroke.

## 5. Conclusions

In conclusion, this study demonstrates that both the CHG index and the RCII index are independently associated with poor functional outcomes, larger infarct size, and—specifically for RCII—hemorrhagic transformation in young patients with acute ischemic stroke. These readily calculable composite biomarkers capture the interplay between metabolic dysregulation and systemic inflammation, providing valuable prognostic information beyond traditional risk factors. These indices may serve as complementary tools for prognostic assessment in research settings; however, their clinical utility for individualized risk stratification requires further validation in prospective studies.

## Figures and Tables

**Table 1:** Baseline characteristics among groups categorized by the outcome at 90 days.

Characteristics	Overall	Good Outcome	Poor Outcome	*p*-Value
*N*	541	430	111	
Age, years	37.0 (33.0–42.0)	37.0 (32.0–41.0)	38.0 (35.0–42.0)	0.109
BMI, kg/m^2^	26.7 ± 4.1	26.8 ± 4.2	26.4 ± 3.6	0.324
CHG	5.1 ± 0.4	5.1 ± 0.4	5.2 ± 0.4	0.018
RCII	1.8 (0.7–4.4)	1.7 (0.6–4.1)	2.8 (1.1–6.9)	<0.001
CRP	1.0 (0.4–2.4)	0.9 (0.4–2.0)	1.5 (0.8–3.2)	<0.001
NIHSS score	2.0 (0.0–5.0)	1.0 (0.0–3.0)	8.0 (6.0–9.0)	<0.001
Gender, *n* (%)				0.873
Male	453 (83.7)	359 (83.5)	94 (84.7)	
Female	88 (16.3)	71 (16.5)	17 (15.3)	
Diabetes, *n* (%)				0.739
No	450 (83.2)	356 (82.8)	94 (84.7)	
Yes	91 (16.8)	74 (17.2)	17 (15.3)	
Hypertension, *n* (%)				0.831
No	290 (53.6)	232 (54.0)	58 (52.3)	
Yes	251 (46.4)	198 (46.0)	53 (47.7)	
Atrial Fibrillation, *n* (%)				0.608
No	535 (98.9)	426 (99.1)	109 (98.2)	
Yes	6 (1.1)	4 (0.9)	2 (1.8)	
Coronary Heart Disease, *n* (%)				0.551
No	523 (96.7)	414 (96.3)	109 (98.2)	
Yes	18 (3.3)	16 (3.7)	2 (1.8)	
Pulmonary infection, *n* (%)				0.007
No	522 (96.5)	420 (97.7)	102 (91.9)	
Yes	19 (3.5)	10 (2.3)	9 (8.1)	
Previous IS, *n* (%)				0.224
No	487 (90.0)	391 (90.9)	96 (86.5)	
Yes	54 (10.0)	39 (9.1)	15 (13.5)	
Smoking, *n* (%)				0.059
No	265 (49.0)	220 (51.2)	45 (40.5)	
Yes	276 (51.0)	210 (48.8)	66 (59.5)	
Alcohol, *n* (%)				0.372
No	377 (69.7)	304 (70.7)	73 (65.8)	
Yes	164 (30.3)	126 (29.3)	38 (34.2)	
TOAST classification, *n* (%)				0.092
LAA	243 (44.9)	182 (42.3)	61 (55.0)	0.023
CE	36 (6.7)	28 (6.5)	8 (7.2)	0.961
SAO	68 (12.6)	55 (12.8)	13 (11.7)	0.885
OE	115 (21.3)	95 (22.1)	20 (18.0)	0.421
UE	79 (14.6)	70 (16.3)	9 (8.1)	0.043

Poor functional outcome was defined as a 90-day mRS score ≥ 2. Continuous variables with normal distribution are expressed as mean (SD), while non-normally distributed continuous variables are expressed as median (IQR). Categorical variables are presented as *n* (%). BMI, body mass index; IS, ischemic stroke; CHG index, cholesterol, high-density lipoprotein, and glucose index; RCII, remnant cholesterol inflammatory index; CRP, C-reactive protein; NIHSS, National Institutes of Health Stroke Scale; LAA, large-artery atherosclerosis; CE, cardioembolism; SAO, small artery occlusion; OE, other etiology; UE, undetermined etiology.

**Table 2:** Logistic regression models for the association between the CHG Index and clinical outcomes or imaging findings.

	Univariate Analysis		Multivariate Analysis	
	OR (95% CI)	*p*	OR (95% CI)	*p*
**Poor outcome at 90 d**				
Continuous (per SD)	1.28 (1.08–1.53)	0.005	1.31 (1.07–1.61)	0.010
Q1	1 (Reference)			
Q2	1.57 (0.92–2.69)	0.100	1.51 (0.85–2.66)	0.156
Q3	1.47 (0.85–2.52)	0.164	1.43 (0.80–2.56)	0.223
Q4	1.99 (1.18–3.36)	0.010	1.99 (1.09–3.61)	0.025
Trend	1.22 (1.03–1.43)	0.018	1.22 (1.01–1.47)	0.041
**Poor outcome at discharge**				
Continuous (per SD)	1.38 (1.18–1.62)	<0.001	1.32 (1.10–1.59)	0.003
Q1	1 (Reference)			
Q2	1.53 (0.96–2.44)	0.073	1.38 (0.84–2.25)	0.200
Q3	1.45 (0.91–2.32)	0.117	1.27 (0.77–2.08)	0.351
Q4	2.19 (1.39–3.46)	<0.001	1.74 (1.03–2.92)	0.037
Trend	1.26 (1.09–1.45)	0.002	1.17 (0.99–1.37)	0.065
**Hemorrhagic transformation**				
Continuous (per SD)	1.04 (0.84–1.28)	0.714	1.11 (0.87–1.43)	0.385
Q1	1 (Reference)			
Q2	1.25 (0.70–2.23)	0.448	1.32 (0.71–2.42)	0.379
Q3	0.82 (0.44–1.54)	0.540	0.88 (0.45–1.72)	0.713
Q4	1.05 (0.58–1.91)	0.861	1.23 (0.62–2.43)	0.552
Trend	0.97 (0.81–1.18)	0.790	1.02 (0.82–1.27)	0.862
**Lesion size**				
Continuous (per SD)	1.27 (1.10–1.47)	0.001	1.40 (1.18–1.66)	<0.001
Q1	1 (Reference)			
Q2	1.38 (0.90–2.11)	0.139	1.48 (0.95–2.31)	0.085
Q3	1.75 (1.15–2.65)	0.009	1.97 (1.26–3.08)	0.003
Q4	1.86 (1.22–2.83)	0.004	2.28 (1.41–3.69)	<0.001
Trend	1.23 (1.08–1.40)	0.002	1.32 (1.13–1.53)	<0.001

The multivariate models were adjusted for age, gender, BMI, history of hypertension, history of dyslipidemia, history of diabetes mellitus, current smoking, current alcohol consumption, history of CHD, atrial fibrillation, pulmonary infection, intravenous thrombolysis treatment, previous ischemic stroke, admission NIHSS score, and TOAST classification. BMI, body mass index; CHD, coronary heart disease; NIHSS, National Institutes of Health Stroke Scale; CHG index, cholesterol, high-density lipoprotein, and glucose index; OR, odds ratio; CI, confidence interval.

**Table 3:** Logistic regression models for the association between the RCII Index and clinical outcomes or imaging findings.

	Univariate Analysis		Multivariate Analysis	
	OR (95% CI)	*p*	OR (95% CI)	*p*
**Poor outcome at 90 d**				
Continuous (per SD)	1.18 (1.01–1.40)	0.043	1.23 (1.04–1.46)	0.016
Q1	1 (Reference)			
Q2	1.33 (0.73–2.42)	0.350	1.50 (0.81–2.81)	0.199
Q3	1.75 (0.98–3.11)	0.059	1.97 (1.07–3.62)	0.030
Q4	3.22 (1.86–5.58)	<0.001	3.43 (1.90–6.20)	<0.001
Trend	1.48 (1.24–1.76)	<0.001	1.50 (1.25–1.80)	<0.001
**Poor outcome at discharge**				
Continuous (per SD)	1.19 (1.00–1.41)	0.045	1.22 (1.04–1.45)	0.017
Q1	1 (Reference)			
Q2	1.04 (0.63–1.74)	0.871	1.15 (0.67–1.95)	0.618
Q3	1.74 (1.07–2.83)	0.026	1.71 (1.01–2.87)	0.044
Q4	3.21 (1.99–5.17)	<0.001	3.12 (1.86–5.21)	<0.001
Trend	1.51 (1.30–1.76)	<0.001	1.48 (1.26–1.75)	<0.001
**Hemorrhagic transformation**				
Continuous (per SD)	1.23 (1.03–1.45)	0.019	1.26 (1.05–1.51)	0.011
Q1	1 (Reference)			
Q2	1.27 (0.65–2.47)	0.485	1.33 (0.66–2.67)	0.419
Q3	1.27 (0.65–2.47)	0.485	1.48 (0.73–3.00)	0.278
Q4	1.98 (1.06–3.70)	0.033	2.42 (1.23–4.75)	0.011
Trend	1.24 (1.01–1.51)	0.037	1.33 (1.07–1.65)	0.010
**Lesion size**				
Continuous (per SD)	1.20 (1.01–1.41)	0.034	1.23 (1.04–1.46)	0.018
Q1	1 (Reference)			
Q2	1.56 (1.00–2.43)	0.049	1.71 (1.07–2.73)	0.025
Q3	1.72 (1.10–2.70)	0.017	1.87 (1.15–3.03)	0.011
Q4	2.84 (1.83–4.41)	<0.001	3.43 (2.12–5.52)	<0.001
Trend	1.38 (1.20–1.59)	<0.001	1.46 (1.26–1.70)	<0.001

The multivariate models were adjusted for age, gender, BMI, history of hypertension, history of dyslipidemia, history of diabetes mellitus, current smoking, current alcohol consumption, history of CHD, atrial fibrillation, pulmonary infection, intravenous thrombolysis treatment, previous ischemic stroke, admission NIHSS score, and TOAST classification. BMI, body mass index; CHD, coronary heart disease; NIHSS, National Institutes of Health Stroke Scale; RCII, remnant cholesterol inflammatory index; OR, odds ratio; CI, confidence interval.

**Table 4:** Incremental discriminative performance of the CHG index and RCII index for discriminating between patients with good and poor 90-day functional outcomes.

Indicators	AUC (95% CI)	NRI	IDI	*p*-Value for DeLong Test
base model	0.564 (0.515–0.619)	0.148 (0.065–0.234)	0.008 (0.001–0.017)	0.050
base model + CHG	0.600 (0.551–0.648)			
base model + RCII	0.645 (0.595–0.695)	0.286 (0.138–0.428)	0.035 (0.020–0.049)	0.004

The base model includes age, gender, BMI, history of hypertension, history of dyslipidemia, history of diabetes mellitus, current smoking, current alcohol consumption, history of CHD, atrial fibrillation, pulmonary infection, intravenous thrombolysis treatment, previous ischemic stroke, admission NIHSS score, and TOAST classification. CHG index, cholesterol, high-density lipoprotein, and glucose index; RCII, remnant cholesterol inflammatory index; BMI, body mass index; CHD, coronary heart disease; NIHSS, National Institutes of Health Stroke Scale; AUC, area under the curve; IDI, integrated discrimination improvement; NRI, net reclassification improvement; CI, confidence interval.

**Table 5:** Sensitivity analysis using mRS ≥ 3 as the threshold for poor functional outcome.

	Univariate Analysis		Multivariate Analysis	
	**OR (95% CI)**	***p***	**OR (95% CI)**	***p***
**Poor outcome at 90 d** **(mRS ≥ 3)**				
**CHG**				
Continuous (per SD)	1.18 (0.93–1.51)	0.178	1.19 (0.90–1.58)	0.231
Q1	1 (Reference)			
Q2	2.41 (1.10–5.28)	0.028	2.50 (1.07–5.82)	0.034
Q3	1.45 (0.62–3.37)	0.389	1.64 (0.66–4.09)	0.289
Q4	2.27 (1.03–4.99)	0.042	2.57 (1.02–6.45)	0.044
Trend	1.18 (0.94–1.49)	0.150	1.22 (0.93–1.60)	0.143
**RCII**				
Continuous (per SD)	1.14 (0.95–1.38)	0.167	1.23 (1.00–1.51)	0.046
Q1	1 (Reference)			
Q2	1.13 (0.44–2.85)	0.802	1.40 (0.53–3.69)	0.491
Q3	1.88 (0.80–4.39)	0.145	2.19 (0.88–5.40)	0.090
Q4	4.20 (1.93–9.14)	<0.001	5.20 (2.24–12.08)	<0.001
Trend	1.70 (1.33–2.19)	<0.001	1.79 (1.37–2.33)	<0.001

The multivariate models were adjusted for age, gender, BMI, history of hypertension, history of dyslipidemia, history of diabetes mellitus, current smoking, current alcohol consumption, history of CHD, atrial fibrillation, pulmonary infection, intravenous thrombolysis treatment, previous ischemic stroke, admission NIHSS score, and TOAST classification. BMI, body mass index; CHD, coronary heart disease; NIHSS, National Institutes of Health Stroke Scale; RCII, remnant cholesterol inflammatory index; OR, odds ratio; CI, confidence interval.

**Table 6:** Sensitivity analysis after excluding patients with pulmonary infection.

	Univariate Analysis		Multivariate Analysis	
	**OR (95% CI)**	***p***	**OR (95% CI)**	***p***
**Poor outcome at 90 d** **(mRS ≥ 3)**				
**CHG**				
Continuous (per SD)	1.16 (0.89–1.51)	0.279	1.13 (0.83–1.55)	0.432
Q1	1 (Reference)			
Q2	2.57 (1.09–6.08)	0.031	2.52 (0.99–6.45)	0.054
Q3	1.70 (0.68–4.22)	0.256	1.72 (0.64–4.63)	0.284
Q4	2.12 (0.88–5.11)	0.095	2.24 (0.80–6.22)	0.123
Trend	1.15 (0.90–1.48)	0.258	1.16 (0.87–1.56)	0.311
**RCII**				
Continuous (per SD)	1.10 (0.89–1.36)	0.377	1.18 (0.92–1.50)	0.186
Q1	1 (Reference)			
Q2	1.27 (0.49–3.31)	0.627	1.53 (0.56–4.16)	0.406
Q3	1.70 (0.68–4.22)	0.256	1.86 (0.71–4.90)	0.210
Q4	3.55 (1.55–8.15)	0.003	3.88 (1.56–9.66)	0.004
Trend	1.56 (1.20–2.03)	<0.001	1.57 (1.18–2.09)	0.002

The multivariate models were adjusted for age, gender, BMI, history of hypertension, history of dyslipidemia, history of diabetes mellitus, current smoking, current alcohol consumption, history of CHD, atrial fibrillation, intravenous thrombolysis treatment, previous ischemic stroke, admission NIHSS score, and TOAST classification. BMI, body mass index; CHD, coronary heart disease; NIHSS, National Institutes of Health Stroke Scale; RCII, remnant cholesterol inflammatory index; OR, odds ratio; CI, confidence interval.

## Data Availability

The datasets used and/or analyzed during the current study are available from the corresponding author upon reasonable request.

## Supplementary Materials

The following supporting information can be downloaded at: https://www.mdpi.com/article/10.3390/jcm15114327/s1, Figure S1. Distribution of continuous demographic and clinical variables; Table S1. Baseline characteristics and clinical features of patients with hemorrhagic transformation.

## Abbreviations

The following abbreviations are used in this manuscript:

AGEs	Advanced Glycation End-products
AUC	Area Under the Curve
BMI	Body Mass Index
CE	Cardioembolism
CHARLS	China Health and Retirement Longitudinal Study
CHD	Coronary Heart Disease
CHG	Cholesterol, High-Density Lipoprotein, and Glucose
CI	Confidence Interval
CRP	C-reactive Protein
FBG	Fasting Blood Glucose
GBD	Global Burden of Disease
HDL-C	High-Density Lipoprotein Cholesterol
HOMA-IR	Homeostatic Model Assessment of Insulin Resistance
IDI	Integrated Discrimination Improvement
IQR	Interquartile Range
IR	Insulin Resistance
LAA	Large-Artery Atherosclerosis
LDL-C	Low-Density Lipoprotein Cholesterol
mRS	Modified Rankin Scale
NIHSS	National Institutes of Health Stroke Scale
NRI	Net Reclassification Improvement
OE	Other Etiology
OR	Odds Ratio
RC	Remnant Cholesterol
RCII	Remnant Cholesterol Inflammatory Index
RCS	Restricted Cubic Spline
ROC	Receiver Operating Characteristic
SAO	Small Artery Occlusion
SD	Standard Deviation
TC	Total Cholesterol
TIA	Transient Ischemic Attack
UE	Undetermined Etiology

## References

[1] Ma Z., He W., Zhou Y., Mai L., Xu L., Li C., Li M. (2024). Global Burden of Stroke in Adolescents and Young Adults (Aged 15–39 Years) from 1990 to 2019: A Comprehensive Trend Analysis Based on the Global Burden of Disease Study 2019. BMC Public Health.

[2] GBD 2021 Global Sepsis Collaborators (2025). Global, Regional, and National Sepsis Incidence and Mortality, 1990–2021: A systematic analysis. Lancet Glob. Health.

[3] Steinberger J., Daniels S.R., Hagberg N., Isasi C.R., Kelly A.S., Lloyd-Jones D., Pate R.R., Pratt C., Shay C.M., Towbin J.A. (2016). Cardiovascular Health Promotion in Children: Challenges and Opportunities for 2020 and Beyond: A Scientific Statement from the American Heart Association. Circulation.

[4] Umer A., Kelley G.A., Cottrell L.E., Giacobbi P., Innes K.E., Lilly C.L. (2017). Childhood Obesity and Adult Cardiovascular Disease Risk Factors: A Systematic Review with Meta-Analysis. BMC Public Health.

[5] Chen R., Ovbiagele B., Feng W. (2016). Diabetes and Stroke: Epidemiology, Pathophysiology, Pharmaceuticals and Outcomes. Am. J. Med. Sci..

[6] DeFronzo R.A., Ferrannini E. (1991). Insulin Resistance: A Multifaceted Syndrome Responsible for NIDDM, Obesity, Hypertension, Dyslipidemia, and Atherosclerotic Cardiovascular Disease. Diabetes Care.

[7] Muniyappa R., Montagnani M., Koh K.K., Quon M.J. (2007). Cardiovascular Actions of Insulin. Endocr. Rev..

[8] Matthews D.R., Hosker J.P., Rudenski A.S., Naylor B.A., Treacher D.F., Turner R.C. (1985). Homeostasis Model Assessment: Insulin Resistance and ?-Cell Function from Fasting Plasma Glucose and Insulin Concentrations in Man. Diabetologia.

[9] Wallace T.M., Levy J.C., Matthews D.R. (2004). Use and Abuse of HOMA Modeling. Diabetes Care.

[10] Mo D., Zhang P., Zhang M., Dai H., Guan J. (2025). Cholesterol, High-Density Lipoprotein, and Glucose Index versus Triglyceride–Glucose Index in Predicting Cardiovascular Disease Risk: A Cohort Study. Cardiovasc. Diabetol..

[11] Tang J., Wei S., Li Y., Zhou Y. (2025). Assessment of Cholesterol-HDL-Glucose Index in Anticipating Risk of Cardiometabolic Diseases: A Comparative Study with Triglyceride-Glucose Index. Sci. Rep..

[12] Chen J., Wu Q., Liu H., Hu W., Zhu J., Ji Z., Yin J. (2025). Predictive Value of Remnant Cholesterol Inflammatory Index for Stroke Risk: Evidence from the China Health and Retirement Longitudinal Study. J. Adv. Res..

[13] Yu Y., Zhang Y., Zhu C., Duan T., Rao Z. (2025). Remnant Cholesterol Inflammatory Index, Calculated from Residual Cholesterol to C-Reactive Protein Ratio, and Stroke Outcomes: A Retrospective Study Using the National Institutes of Health Stroke Scale and Modified Rankin Scale. Lipids Health Dis..

[14] Adams H.P., Bendixen B.H., Kappelle L.J., Biller J., Love B.B., Gordon D.L., Marsh E.E. (1993). Classification of Subtype of Acute Ischemic Stroke. Definitions for Use in a Multicenter Clinical Trial. TOAST. Trial of Org 10172 in Acute Stroke Treatment. Stroke.

[15] Muscari A., Faccioli L., Lega M.V., Lorusso A., Masetti M., Pastore Trossello M., Puddu G.M., Spinardi L., Zoli M. (2019). Predicting Hemorrhagic Transformation and Its Timing from Maximum Cerebral Lesion Diameter in Nonlacunar Ischemic Strokes. Brain Behav..

[16] Montellano F.A., Ungethüm K., Ramiro L., Nacu A., Hellwig S., Fluri F., Whiteley W.N., Bustamante A., Montaner J., Heuschmann P.U. (2021). Role of Blood-Based Biomarkers in Ischemic Stroke Prognosis. Stroke.

[17] Soldozy S., Yağmurlu K., Norat P., Elsarrag M., Costello J., Farzad F., Sokolowski J.D., Sharifi K.A., Elarjani T., Burks J. (2022). Biomarkers Predictive of Long-Term Outcome After Ischemic Stroke: A Meta-Analysis. World Neurosurg..

[18] Ginsberg H.N. (2000). Insulin Resistance and Cardiovascular Disease. J. Clin. Investig..

[19] Beckman J.A., Creager M.A., Libby P. (2002). Diabetes and Atherosclerosis. JAMA.

[20] Giacco F., Brownlee M. (2010). Oxidative Stress and Diabetic Complications. Circ. Res..

[21] Tabit C.E., Chung W.B., Hamburg N.M., Vita J.A. (2010). Endothelial Dysfunction in Diabetes Mellitus: Molecular Mechanisms and Clinical Implications. Rev. Endocr. Metab. Disord..

[22] Yang C., Ma M., Jiang X., Zhang L., Zhang Y., Zhou M., He L., Fang J. (2025). Association of the CHG Index with 90-Day Functional Outcomes and Mortality in Acute Ischemic Stroke after Endovascular Therapy: A Retrospective Study. J. Stroke Cerebrovasc. Dis..

[23] Huang L., Li L., Yu M., Xu L. (2025). A Comparative Analysis of the Cholesterol-High-Density Lipoprotein-Glu Cose Index and the Triglyceride-Glucose Index in Predicting in-Hospita l Mortality in Critically Ill Ischemic Stroke Patients. Front. Neurol..

[24] Whiteley W., Chong W.L., Sengupta A., Sandercock P. (2009). Blood Markers for the Prognosis of Ischemic Stroke. Stroke.

[25] Di Napoli M., Schwaninger M., Cappelli R., Ceccarelli E., Di Gianfilippo G., Donati C., Emsley H.C.A., Forconi S., Hopkins S.J., Masotti L. (2005). Evaluation of C-Reactive Protein Measurement for Assessing the Risk and Prognosis in Ischemic Stroke. Stroke.

[26] Bang O.Y., Saver J.L., Buck B.H., Alger J.R., Starkman S., Ovbiagele B., Kim D., Jahan R., Duckwiler G.R., Yoon S.R. (2007). Impact of Collateral Flow on Tissue Fate in Acute Ischaemic Stroke. J. Neurol. Neurosurg. Psychiatry.

[27] Doi T., Langsted A., Nordestgaard B.G. (2022). Elevated Remnant Cholesterol Reclassifies Risk of Ischemic Heart Disea Se and Myocardial Infarction. J. Am. Coll. Cardiol..

[28] Li D., Xu Z., Wang F., Hu Y., Zhang X., Yang J., Wan Q., Zhang N., Liu Y. (2025). The Role of Three Glucose/Lipid Composite Indices (CHG, TYG, and AIP) in Predicting Carotid Plaque and Fatty Liver Outcomes: A Retrospective Cohort Study. Front. Endocrinol..

[29] Paneni F., Costantino S., Cosentino F. (2015). Role of Oxidative Stress in Endothelial Insulin Resistance. World J. Diabetes.

[30] Tangvarasittichai S. (2015). Oxidative Stress, Insulin Resistance, Dyslipidemia and Type 2 Diabetes Mellitus. World J. Diabetes.

[31] Wang K., Wang R., Yang J., Liu X., Shen H., Sun Y., Zhou Y., Fang Z., Ge H. (2022). Remnant Cholesterol and Atherosclerotic Cardiovascular Disease: Metabo Lism, Mechanism, Evidence, and Treatment. Front. Cardiovasc. Med..

[32] Raza A., Saleem S., Imran S., Rahman S., Haroon M., Razzaq A., Hussain A., Iqbal J., Sathian B. (2025). From Metabolic Dysregulation to Neurodegenerative Pathology: The Role of Hyperglycemia, Oxidative Stress, and Blood-Brain Barrier Breakdown in T2D-Driven Alzheimer’s Disease. Metab. Brain Dis..

[33] Ding P.-F., Zhang H.-S., Wang J., Gao Y.-Y., Mao J.-N., Hang C.-H., Li W. (2022). Insulin Resistance in Ischemic Stroke: Mechanisms and Therapeutic Approaches. Front. Endocrinol..

[34] Honig A., Percy J., Sepehry A.A., Gomez A.G., Field T.S., Benavente O.R. (2022). Hemorrhagic Transformation in Acute Ischemic Stroke: A Quantitative Systematic Review. J. Clin. Med..

[35] Hong J.M., Kim D.S., Kim M. (2021). Hemorrhagic Transformation After Ischemic Stroke: Mechanisms and Manag Ement. Front. Neurol..

[36] Jampathong N., Laopaiboon M., Rattanakanokchai S., Pattanittum P. (2018). Prognostic Models for Complete Recovery in Ischemic Stroke: A Systematic Review and Meta-Analysis. BMC Neurol..

[37] Karakasis P., Patoulias D., Rizzo M., Fragakis N., Mantzoros C.S. (2025). Association between Remnant Cholesterol and Chronic Kidney Disease: Systematic Review and Meta-Analysis. Diabetes Obes. Metab..

