# Supplementary material for: Prognostic Value of the Cholesterol, High-Density Lipoprotein, and Glucose Index and Remnant Cholesterol Inflammatory Index in Young Patients with Acute Ischemic Stroke
Source: J Clin Med. 2026 Jun 3;15(11):4327. doi: 10.3390/jcm15114327 (PMC13258509; doi:10.3390/jcm15114327)
Supplement: Supplementary file 1 [file jcm-15-04327-s001.zip › jcm-4298439-supplementary.pdf]

Supplementary Figure S1. Distribution of continuous demographic and clinical variables.

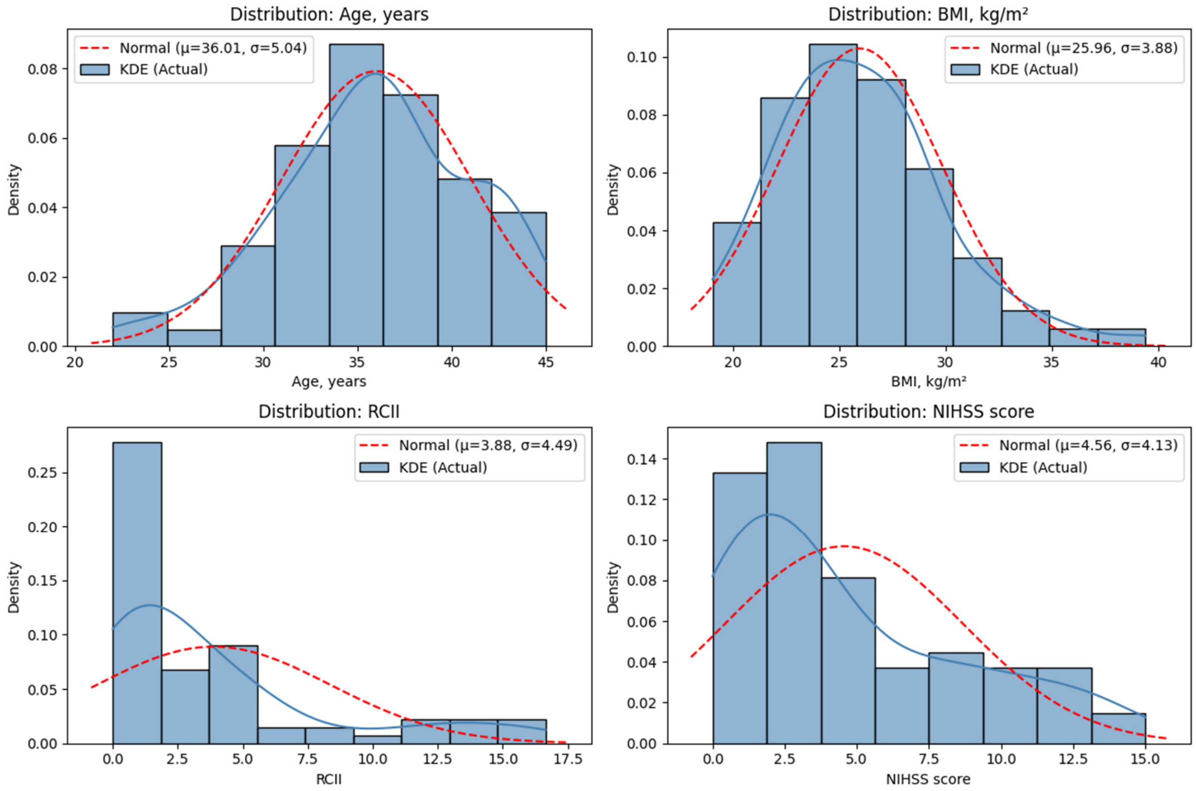

The panels display the probability density histograms for Age (top left), Body Mass Index (BMI, top right), RCII (bottom left), and National Institutes of Health Stroke Scale (NIHSS) score (bottom right). Within each panel, the solid line indicates the Kernel Density Estimation (KDE) of the actual data, while the dashed line represents the fitted theoretical normal distribution.

Supplementary Table S1. Baseline characteristics and clinical features of patients with hemorrhagic transformation.

Characteristics	Overall	Asymptomatic	Symptomatic	P value
N	71	54	17	
**Age, years**	36.0 (33.0-39.0)	36.0 (32.0-38.8)	37.0 (35.0-41.0)	0.156
**BMI, kg/m²**	26.0 ± 3.9	26.2 ± 3.8	25.3 ± 4.3	0.41
**RCII**	1.8 (0.9-4.9)	1.6 (0.9-4.8)	2.9 (1.0-6.2)	0.263
**NIHSS score**	3.0 (1.5-7.5)	4.0 (2.0-8.0)	2.0 (1.0-4.0)	0.16
**Gender, n (%)**				0.67
Male	63 (88.7)	47 (87.0)	16 (94.1)	
Female	8 (11.3)	7 (13.0)	1 (5.9)	
**Diabetes, n (%)**				0.143
No	59 (83.1)	47 (87.0)	12 (70.6)	

Yes	12 (16.9)	7 (13.0)	5 (29.4)	
**Hypertension, n (%)**				0.644
No	49 (69.0)	36 (66.7)	13 (76.5)	
Yes	22 (31.0)	18 (33.3)	4 (23.5)	
**Smoking, n (%)**				0.142
No	34 (47.9)	29 (53.7)	5 (29.4)	
Yes	37 (52.1)	25 (46.3)	12 (70.6)	
**Alcohol, n (%)**				0.874
No	45 (63.4)	35 (64.8)	10 (58.8)	
Yes	26 (36.6)	19 (35.2)	7 (41.2)	
**Endovascular treatment, n (%)**				0.04
No	67 (94.4)	53 (98.1)	14 (82.4)	
Yes	4 (5.6)	1 (1.9)	3 (17.6)	
**Antithrombotic treatment, n (%)**				0.08
SAPT	31 (43.7)	20 (37.0)	11 (64.7)	0.084
DAPT	35 (49.3)	31 (57.4)	4 (23.5)	0.031
SAPT + Anticoagulation	2 (2.8)	1 (1.9)	1 (5.9)	0.424
DAPT + Anticoagulation	1 (1.4)	1 (1.9)	0 (0.0)	NA
Anticoagulation only	1 (1.4)	1 (1.9)	0 (0.0)	NA
No Antithrombotic treatment	1 (1.4)	0 (0.0)	1 (5.9)	0.239
**ECASS classification, n (%)**				<0.001
HI1	40 (56.3)	39 (72.2)	1 (5.9)	<0.001
HI2	22 (31.0)	14 (25.9)	8 (47.1)	0.179
PH1	7 (9.9)	1 (1.9)	6 (35.3)	<0.001
PH2	2 (2.8)	0 (0.0)	2 (11.8)	0.055

Among patients with hemorrhagic transformation, the distribution of ECASS subtypes, reperfusion therapy use, and antithrombotic medication exposure are summarized based on symptomatic status. Continuous variables are expressed as mean ± standard deviation or median (interquartile range), depending on the data distribution. Categorical variables are presented as frequencies (percentages). BMI, Body Mass Index; RCII, remnant cholesterol inflammatory index; NIHSS, National Institutes of Health Stroke Scale; SAPT, Single Antiplatelet Therapy; DAPT, Dual Antiplatelet Therapy; ECASS, European Cooperative Acute Stroke Study; HI, Hemorrhagic Infarction; PH, Parenchymal Hemorrhage; NA, Not Applicable.
